# Atrioventricular nodal reentrant tachycardia onset, sustainability, and spontaneous termination in rabbit atrioventricular node model with autonomic nervous system control

**DOI:** 10.3389/fphys.2024.1529426

**Published:** 2025-01-17

**Authors:** Maxim Ryzhii, Elena Ryzhii

**Affiliations:** ^1^ Department of Computer Science, University of Aizu, Aizu-Wakamatsu, Japan; ^2^ Department of Anatomy and Histology, Fukushima Medical University, Fukushima, Japan

**Keywords:** atrioventricular node, rabbit heart model, Aliev-Panfilov model, dual pathway, autonomic nervous system, AVNRT, effective refractory period, computer simulation

## Abstract

Atrioventricular nodal reentrant tachycardia (AVNRT) is one of the most common types of paroxysmal supraventricular tachycardia. The activity of the autonomic nervous system (ANS) is known to influence episodes of AVNRT, yet the precise mechanisms underlying this effect remain incompletely understood. In this study, we update our compact multifunctional model of the rabbit atrioventricular (AV) node with ANS control to simulate AVNRT. The refractoriness of the model cells is adjusted by a specific ANS coefficient, which impacts the effective refractory periods, conduction delays, and intrinsic frequency of pacemaker cells. Using this model, we investigate the onset, sustainability, and spontaneous termination of typical slow-fast and atypical fast-slow forms of AVNRT under ANS modulation. The conditions for the onset and sustainability of AVNRT can exist independently in various combinations. Differences in the effective refractory periods of the slow and fast pathways of the AV node during anterograde and retrograde conduction determine the specific form of AVNRT. For the first time, a computer model reveals the potential to identify hidden processes within the AV node, thereby bringing us closer to understanding the role of ANS control in AVNRT. The results obtained are consistent with clinical and experimental data and represent a novel tool for studying the electrophysiological mechanisms behind this type of arrhythmia.

## 1 Introduction

The atrioventricular (AV) node consists of dual pathways: a fast pathway (FP) with a relatively longer effective refractory period (ERP) and a slow pathway (SP) with a shorter ERP. These pathways can create a reentrant circuit, a substrate for AV nodal reentrant tachycardia (AVNRT), the most common type among regular supraventricular arrhythmias (Straus and Schocken, 2021).

AVNRT manifests as sudden episodes of a few cycles of abnormally fast heartbeats (reciprocal or echo beats) or as sustained or persistent tachycardia. AVNRT is electrophysiologically classified as typical (slow-fast) and atypical (fast-slow and slow-slow) forms corresponding to anterograde-retrograde conduction sequence through the dual AV nodal pathways (Katritsis and Josephson, 2013).

The autonomic nervous system (ANS) plays a crucial role in the initiation and termination of supraventricular tachycardias within the AV node (Nigro et al., 2010). Sympathetic stimulation typically facilitates the induction of AVNRT, while enhancing vagal (parasympathetic) tone via pharmacological means or Valsalva maneuvers is commonly employed to terminate the tachycardia (Appelboam et al., 2015; Xiao et al., 2024). The effect of ANS control on dual pathways interaction in the initiation, sustainability, and spontaneous termination of AVNRT is still poorly understood despite some attempts to explain its exact underlying physiological mechanism.

Several functional computer models of the AV node have been developed (Inada et al., 2009; Climent et al., 2011; Plappert et al., 2022). However, to our knowledge, only one incorporates ANS control (Plappert et al., 2022). In the latter model, the authors modulate vagal tone by modifying parameters of AV node refractoriness and conduction velocity separately. Recently, we have developed a compact, multi-functional rabbit AV node model based on the simplified two-variable cardiac cell model (Ryzhii and Ryzhii, 2023a). The one-dimensional model is fitted to existing experimental data and includes dual pathway physiology, a primary pacemaker in the sinus node (SN), and a secondary pacemaker in the SP. Visualization of interactions between intact and post-ablated SP and FP in the form of Lewis ladder diagrams facilitates the study of AVNRT.

Experimental observations show that FP has a significantly longer effective refractory period (ERP) in the case of anterograde conduction ( ${\text{aERP}}_{FP}$ ) than that of SP ( ${\text{aERP}}_{SP}$ ), which is a substrate for typical slow-fast AVNRT at stimulation periods shorter than the ${\text{aERP}}_{FP}$ (Reid et al., 2003). This substantial property of normal AV node behavior, demonstrated by simulation and experimental studies (Inada et al., 2009; Climent et al., 2011; Billette and Tadros, 2019), provides effective conduction slowing and fast rhythm filtering. In contrast, in the case of retrograde conduction with His bundle pacing no apparent difference between retrograde ERPs of SP ( ${\text{rERP}}_{SP}$ ) and FP ( ${\text{rERP}}_{FP}$ ) was observed in both control and post-ablation cases. Taking into account the statistical uncertainty of the difference between ${\text{rERP}}_{SP}$ and ${\text{rERP}}_{FP}$ (Reid et al., 2003), in our preliminary work (Ryzhii and Ryzhii, 2023b), we assumed that any relationship between ${\text{rERP}}_{SP}$ and ${\text{rERP}}_{FP}$ values may exist within a reasonable range. We showed that ${\text{rERP}}_{SP}$ and ${\text{rERP}}_{FP}$ affected insignificantly the anterograde conduction in the AV node. Along with the typical AVNRTinduced spontaneously or with pacing maneuvers via the atria, we simulated slow-fast and fast-slow AVNRT forms with His bundle pacing and demonstrated that the difference in aERP and rERP of FP and SP determines the specific form of AVNRT. However, these results did not take into consideration the influence of ANS control.

To overcome this drawback, we updated the functionality of our AV node model incorporating the ANS control. The control by both sympathetic and parasympathetic parts of the ANS was achieved by introducing a single coefficient to scale parameters related to the refractoriness of the model cells. This coefficient also influences other key properties of the cardiac conduction system, including the intrinsic rates of pacemakers and conduction times.

In the current work, we used the modified model to study the onset, susceptibility, and spontaneous termination of typical slow-fast and atypical fast-slow forms of AVNRT. We consider the induction of AVNRT not only by premature atrial and His bundle stimulation, referred to in clinical practice as premature atrial (PAC) and ventricular (PVC) complexes but also by electrical impulses originating within the AVjunction (premature junctional complex, PJC) (Straus and Schocken, 2021).

## 2 Model and methods

**FIGURE 1:**
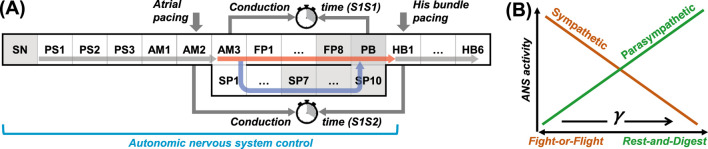
**(A)** Schematic representation of the rabbit atrioventricular node model. SN - sinus node, PS - peripheral sinus node cells, AM - atrial muscle cells, FP - fast pathway cells, SP - slow pathway cells, PB - penetrating bundle cell, HB - His bundle cells. Thick vertical arrows denote places of stimuli application for atrial and His bundle pacing. The arrows within the structure correspond to normal conduction. The gray shading indicates pacemaker cells. **(B)** Relation between ANS activity and the coefficient γ .

The scheme of the compact AV node model used in this study is shown in Figure 1A. Each model cell is described by Aliev-Panfilov cardiac cell model (Aliev and Panfilov, 1996) given by a couple of reaction-diffusion type ordinary differential equations

$$\dot{V}=c\left[kV\left(V-a_{1}\right)\left(1-V\right)-rV\right]+I^{\mathrm{coupl}}+I^{\mathrm{stim}},$$ (1)

$$\dot{r}=c\epsilon \left[-r-kV\left(V-a_{2}-1\right)\right],\;\epsilon =\epsilon_{0}+r\mu_{1}/\left(V+\mu_{2}\right),\;$$ (2)

where V is the dimensionless transmembrane potential, r is the gate variable, c is the time scaling coefficient, and k is the parameter controlling the magnitude of the transmembrane current. Parameters $\epsilon_{0}$ , $a_{1}$ , $a_{2}$ , $\mu_{1}$ , and $\mu_{2}$ determine the conduction characteristics of tissue, $a_{1}>0$ represent the excitation threshold of quiescent excitable cells, while $a_{1}<0$ sets the intrinsic oscillation frequency of the pacemaking cells (gray-shaded in Figure 1A) (Ryzhii and Ryzhii, 2022). The intercellular coupling terms

$$I_{i}^{\mathrm{coupl}}=d_{i-1}\left(V_{i-1}-\beta_{i-1}V_{i}\right)+d_{i}\left(-V_{i}+\beta_{i}V_{i+1}\right)$$

in the one-dimensional system account for the coupling asymmetry, where $d_{i}$ are the diffusion coefficients (normalized on dimensionless distance), i denotes the index of neighboring cells, and the coefficients β<1 correspond to the accelerated anterograde and slowed retrograde conduction, and *vice versa* for β>1 . $I^{\mathrm{stim}}$ denotes external stimulation current applied to the atria or His bundle (thick arrows in Figure 1A) to perform S1S2 and S1S1 stimulation protocols.

Standard S1S2 stimulation consisted of nine pulses with constant basic S1–S1 interval equal to spontaneous sinus rhythm interval determined by the current ANS state, and S2 premature test stimulus with S1–S2 interval introduced with a decrement of 1 ms until conduction through AV node is blocked. The S1S2 conduction time was measured betweenatrial muscle cell AM2 and His bundle cell HB1. S1S1 stimulation was performed by applying ten pulses with 1 ms interval decrement starting from the interval of spontaneous sinus rhythm in the current state of the ANS. For this stimulation type, we measured atria-His and His-atria conduction delays within the AV node ringbetween atrial muscle cell AM3 and penetrating bundle cell PB. Atrial and His bundle stimulation pulses were 1 ms and 2 ms long, respectively, and 1.3 times above the threshold.

We implemented the effect of the ANS in our rabbit cardiac conduction system model by introducing a control coefficient γ (Figure 1B), allowing for dynamic changes of the parameters $\mu_{1}$ and $\mu_{2}$ in Equation 2 that regulate cell refractoriness (Aliev and Panfilov, 1996) :

$$\mu_{1}^{*}=\mu_{1}/\gamma ,\;\mu_{2}^{*}=\mu_{2}\gamma ,\;\epsilon =\epsilon_{0}+r\mu_{1}^{*}/\left(V+\mu_{2}^{*}\right).$$

The rationale for this method of ANS control was discussed in detail in our recent report (Ryzhii and Ryzhii, 2024).The value of the coefficient γ was the same in the model cells from the SN to the PB (Figure 1A), including the AV junctional pacemaker cells (Fedorov et al., 2011), and varied simultaneously, reflecting the effect of a specific ANS state. In the remaining cells of the model, the gamma value was fixed and set to one.

Since only isolated rabbit heart preparations were used in the experiments (Reid et al., 2003; Climent et al., 2011; Billette and Tadros, 2019), any influence of the ANS was absent, leaving the hearts in a state representing a static invariable situation regarding the cardiac conduction system. At such conditions, the onset of AVNRT was observed in the S1S2 protocol stimulation at short atrial test pulses with a normal sinus rhythm of about 166 bpm (360 ms beating interval) (Reid et al., 2003). However, it is known that enhancing sympathetic tone may provoke AVNRT onset (Hartikainen et al., 1997). At the same time, Valsalva maneuver or adenosine administration (Appelboam et al., 2015; Xiao et al., 2024) causes the vagal tone enhancement, resulting in reduced heart rate and consequent termination of AVNRT. Considering the above facts, we set γ=1.0 corresponding to an augmented sympathetic tone state with high sinus rhythm, allowing induction of some form of AVNRT. An increase of γ first leads to a normal rhythm at γ≃1.7 and then to bradycardia when the vagal tone strongly predominates (γ≃2.0) . A decrease of γ means enhancement of sympathetic tone and, respectively, shortens the refractory period, action potential duration of the affected model cells, ERP of both pathways, reduces nodal conduction time, and increases intrinsic rates of the sinus node and AV nodal pacemakers (Chiou et al., 2003).

The three AV node model variants considered had similar anterograde conduction characteristics ( ${\text{aERP}}_{SP}<$ ${\text{aERP}}_{FP}$ ) but different retrograde conduction properties (Ryzhii and Ryzhii, 2023b):

– The *first model variant* had ${\text{rERP}}_{SP}≃$ ${\text{rERP}}_{FP}$ for enhanced sympathetic tone (increased sinus rates) and at normal condition;
– The *second model variant* had slightly reduced diffusion coefficient d for the last three FP cells and increased d for the last four SP cells compared with the first variant which provides longer ${\text{rERP}}_{FP}$ within the entire γ range;
– The *third model variant* had the coupling asymmetry coefficient β reduced five times between SP10 and PB model cells, creating the relationship ${\text{rERP}}_{SP}>$ ${\text{rERP}}_{FP}$ at enhanced sympathetic tone (higher rhythms) and its inversion ( ${\text{rERP}}_{SP}<$ ${\text{rERP}}_{FP}$ ) at normal condition and enhanced vagal tone (reduced rhythms). The refractory period in the proximal part of His bundle (HB1–HB3 cells) was reduced in the second and third model variants compared to the first variant to facilitate His bundle premature stimulation.

For each model variant, we considered three scenarios of premature cardiac complexes classified by their origin - atrial (premature atrial complex, PAC), ventricular or His bundle (premature ventricular complex, PVC), and AV intranodal or junctional (premature junctional complex, PJC).Since our model does not include ventricles, we assume that PVCs originate in the His bundle region, which is observed in clinical practice [see, for example, work by Yamada et al. (2008)]. The cases of PAC and PVC required proper selection of intervals of premature extrastimuli within the AVNRT induction window. For PJC, a short burst of sympathetic activity with high sinus tachycardia was applied to stimulate the conduction block within the AV node.

The ANS coefficient γ was dynamically varied stepwise during the simulations at predefined moments. The intervals between γ changes were selected to reflect the natural reaction of the cardiac conduction to ANS modulation.

The simulations were performed using MATLAB (R2023a, Mathworks Inc., Natick, MA, United States). The ordinary differential Equations 1, 2 were solved using *ode23* solver which utilizes second and third order Runge-Kutta-Fehlberg formulas with automatic step-size. Other parameter values were similar to that in (Ryzhii and Ryzhii, 2023a) and are based on rabbit experimental data (Reid et al., 2003; Billette and Tadros, 2019). Additional details of the basic rabbit AV node model and its properties can be also found in (Ryzhii and Ryzhii, 2023a).

## 3 Results

In what follows, we refer to AVNRT as sustained oscillations within the AV ring and echo beats (reciprocating pulses) as decaying oscillations with with no more than a few cycles.

### 3.1 The first model variant

**FIGURE 2:**
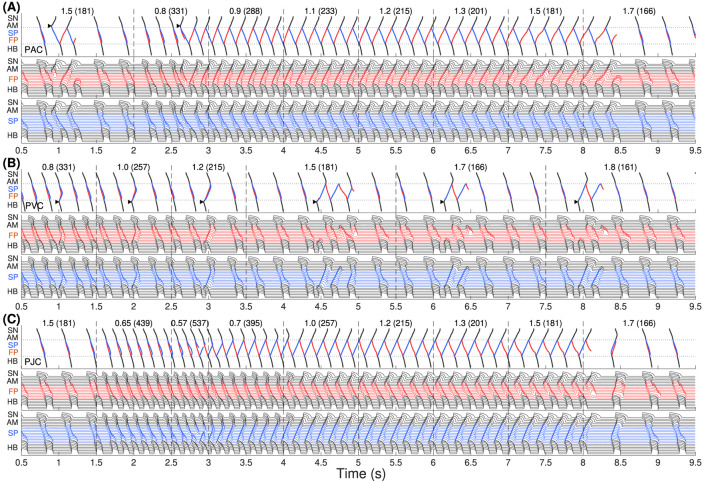
The first model variant with ${\text{rERP}}_{SP}\;≃$ ${\text{rERP}}_{FP}$ . The top panels show the ladder diagrams of excitation propagation in the AV node dual pathway structure from the sinus node (SN) to the His bundle (HB) via fast pathway (FP, red traces) and slow pathway (SP, blue traces). The numbers above the diagrams indicate the value of the coefficient γ and the corresponding sinus rhythm in bpm (in brackets). Black arrowheads denote the places and moments of premature stimulation. The middle and bottom panels demonstrate action potentials passing through the fast and slow pathways. **(A)** Onset of typical slow-fast AVNRT with premature atrial stimulation (premature atrial complex, PAC) at enhanced sympathetic tone followed by spontaneous termination at rising vagal tone. **(B)** No induction of AVNRT was observed with His bundle pacing (premature ventricular complex, PVC). **(C)** A brief burst of very strong sympathetic tone accompanied by an excessively accelerated sinus rhythm (premature junctional complex, PJC) induced atypical fast-slow form of AVNRT followed by spontaneous termination with vagal tone dominance.

Figure 2 demonstrates simulation results of AVNRT onset and spontaneous termination for the first model variant with varying ANS tone (γ) . The top panels demonstrate ladder diagrams of conduction propagation from the sinus node to His bundle via FP (red) and SP (blue color). The value of the current ANS coefficient γ and the corresponding sinus rate in beats per minute (in brackets) are indicated above the ladder diagrams. Vertical dashed lines denote the moments of γ change. The middle and bottom panels show action potential sequences from the sinus node to His bundle separately for FP and SP.

**FIGURE 3:**
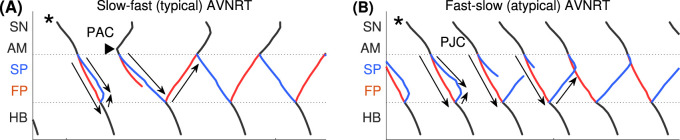
Schemes of AVNRT onset in the first model variant. **(A)** Slow-fast (typical) AVNRT form at the premature atrial complex (PAC) (from Figure 2A), and **(B)** fast-slow (atypical) form at the premature junctional complex (PJC) (from Figure 2C). Arrows show the direction of conduction via pathways. Arrowhead denotes the place and moment of premature stimulation. Asterisk indicates normal conduction.

In Figure 2A, we started with an increased sympathetic tone at γ=1.5 applying premature atrial stimulus (PAC, indicated by black arrowhead), which caused a slow-fast echo beat followed by sinus rhythm. Applying PAC at γ decreased to 0.8, we obtained the onset of typical slow-fast AVNRT. The oscillations persisted with increasing γ up to 1.5 and spontaneously terminated at γ=1.7 with a return to the normal sinus rhythm. Details of slow-fast AVNRT onset with PAC are shown on the ladder diagram in Figure 3A.

With His bundle premature stimulation (PVC), trying to provoke AVNRT at different γ , we obtained only atypical fast-slow echo beats during at γ≥1.5 (Figure 2B).

AVNRT with PJC originates from within the AV node, so it does not require an external premature stimulus. A brief episode with a sudden decrease of γ to the very low value of 0.57, accompanied by a very high sinus rhythm of 537 bpm, resulted in a critical reduction in the duration and amplitude of action potential and block of SP conduction. This triggered fast-slow AVNRT, which persisted with increasing γ until the latter reached a normal value of 1.7 (Figure 2C). Details of fast-slow AVNRT onset of PJC origin are shown on the ladder diagram in Figure 3B. The AVNRT began after retrograde SP excitation met anterograde SP excitation from a subsequent sinus rhythm, and they annihilated each other.

**FIGURE 4:**
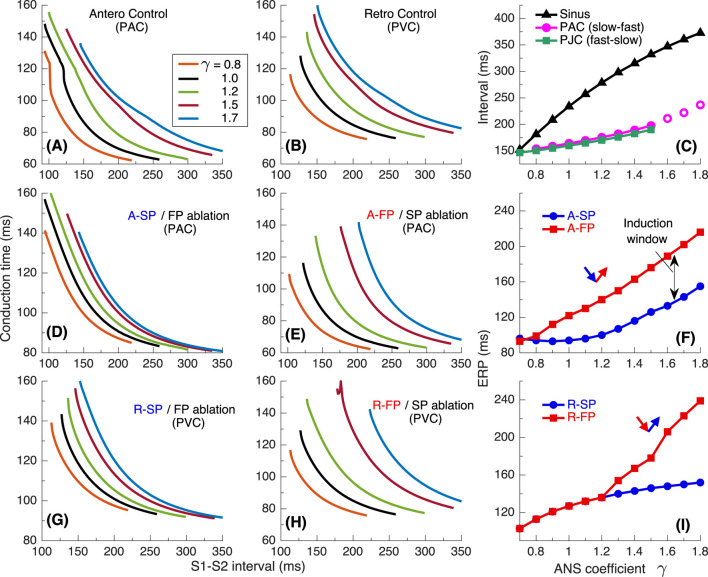
Conduction characteristics using S1S2 stimulation protocol for the first model variant. **(A)** Control case with atrial pacing (PAC). **(B)** Control case with His bundle pacing (PVC). **(C)** Dependence of sinus rhythm and AVNRT intervals of PAC and AV intranodal (PJC) origin on the coefficient γ . Empty markers correspond to echo beats. **(D, E)** - anterograde (A-) conduction times for SP (FP ablation) and FP (SP ablation), and their ERPs **(F–H)** - retrograde (R-) conduction times for SP and FP, and their ERPs **(I)**.

To investigate the underlying physiological background of the results in Figure 2, we performed simulations using S1S2 and S1S1 stimulation protocols. Figure 4 presents various related conduction curves for the first model variant calculated for different ANS states represented by the coefficient γ=0.8 , 1.0, 1.2, 1.5, and 1.7. Figures 4A,B demonstrate anterograde conduction curves with PAC and retrograde conduction curves with PVC for the control (intact) AV node. With decreasing γ , i.e., increasing sympathetic tone, the anterograde conduction switching from FP to SP (Reid et al., 2003; Climent et al., 2011) became more pronounced with sharper tilt (Figure 4A).

Maximal S1-S2 interval values for the conduction curves are limited by spontaneous sinus rhythm interval, which decreases with smaller γ (Figure 4C). Apart from the sinus rhythm interval, ANVRT intervals obtained with PAC and PJC shown in Figures 2A,C are indicated in Figure 4C by filled markers, and echo beats appearing at γ=1.6−1.8 - by open markers. The intervals of AVNRT of different origin are always shorter than the sinus rhythm interval and indicate the overdrive suppression of the latter by the reentrant oscillations.

The S1S2 conduction curves obtained for individual SP and FP pathways (post-ablation cases) and the dependence of their ERPs on γ for PAC and PVC are shown in Figures 4D–I, respectively. The distance between FP-only curves (Figures 4E,H) with changing γ is more pronounced than in the case of SP-only curves (Figures 4D,G) for both atrial and His bundle stimulation. Within the entire useful range of γ , ${\text{aERP}}_{SP}$ is smaller than ${\text{aERP}}_{FP}$ and the incuction window (distance between FP and SP ERP curves) widens with a predominance of parasympathetic tone (Figure 4F). This is common for mammalian AV node (Reid et al., 2003) and creates a possibility of slow-fast AVNRT onset within the wide range of ANS states. On the other hand, the retrograde ERPs of both pathways ( ${\text{rERP}}_{SP}$ and ${\text{rERP}}_{FP}$ ) are equal within the entire range of sympathetic tone, and ${\text{rERP}}_{FP}>$ ${\text{rERP}}_{SP}$ at enhanced parasympathetic tone. (Figure 4I). The above relationships between the SP and FP ERPs allowed the onset of slow-fast AVNRT with atrial pacing at γ≥0.8 (Figure 2A), and blocked the initiation of fast-slow AVNRT with His bundle pacing at γ<1.3 . However, the onset of fast-slow AVNRT or echo beats is possible at γ>1.3 (Figure 2B).

**FIGURE 5:**
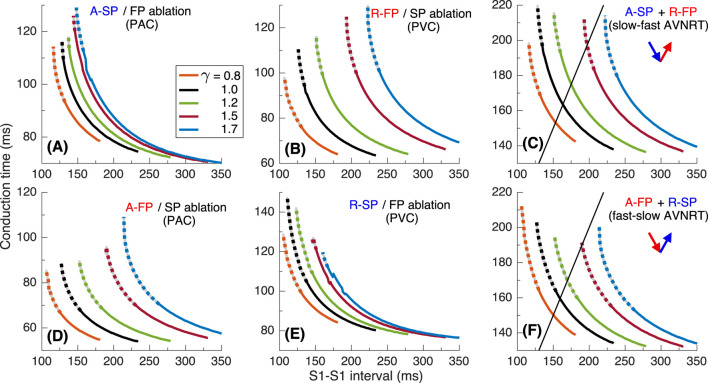
Conduction characteristics using S1S1 stimulation protocol for the first model variant. **(A, B)** - anterograde SP and retrograde FP conduction times and their sum **(C)** *versus* pacing interval. **(D, E)** - anterograde FP and retrograde SP conduction times and their sum **(F)** *versus* pacing interval. Unstable conduction is indicated by thick dotted parts on the conduction curves. Black straight lines in panels **(C, F)** represent the AVNRT sustainability lines on which the stimulation interval equals the sum of the SP and FP conduction times.

Figures 5A–C show the conduction time calculated using S1S1 pacing protocol for anterograde SP, retrograde FP and their sum for slow-fast AVNRT form, and Figures 5D–F - anterograde FP and retrograde SP and their sum for fast-slow form.

In our simulations, the initial time delay preceding the first S1 stimulus of the S1S1 protocol affected the stability of conduction in the pathways. The variation of the initial delay resulted in the unstable conduction of last S1 test stimulus at short S1-S1 intervals [left ends of the conduction curves in Figures 5A,B,D,E. The instability is also reflected in the summation curves in Figures 5C,F. The unstable regions are marked by thick dotted lines of the same color. The earliest (leftmost) point on each summation curve and the width of its unstable region are determined by the largest first unstable point and the largest last unstable point of corresponding conduction curves of either pathway.

AVNRT is a self-sustained oscillation with a cycle length equal to the stimulation period. The AVNRT cycle length equals the sum of the anterograde SP (A-SP) and retrograde FP (R-FP) conduction times for slow-fast AVNRT form and the sum of the anterograde FP (A-FP) and retrograde SP (R-SP) conduction times for fast-slow form (Figures 5C,F). At the same time, the pathway conduction time is cycle-length dependent. The existence of stable periodic oscillations in the AV ring can be determined by the presence of the intersection point of the summation curve with the identity line y=x (where S1-S1 pacing interval equals the sum of SP and FP conduction times), denoted by straight black solid lines (AVNRT sustainability lines) in Figures 5C,F.

As seen from Figures 5C,F, at γ=0.8−1.5 the intersection points between the corresponding summation conduction curves and the AVNRT sustainability lines exist. This indicates the existence of oscillations which may be unstable at γ=1.5 for slow-fast AVNRT type (see also Figure 2A), and at γ=1.2−1.5 for fast-slow type (Figure 2B,C). At γ=1.7 the oscillations cannot persist for both slow-fast and fast-slow AVNRT types.

### 3.2 The second model variant

**FIGURE 6:**
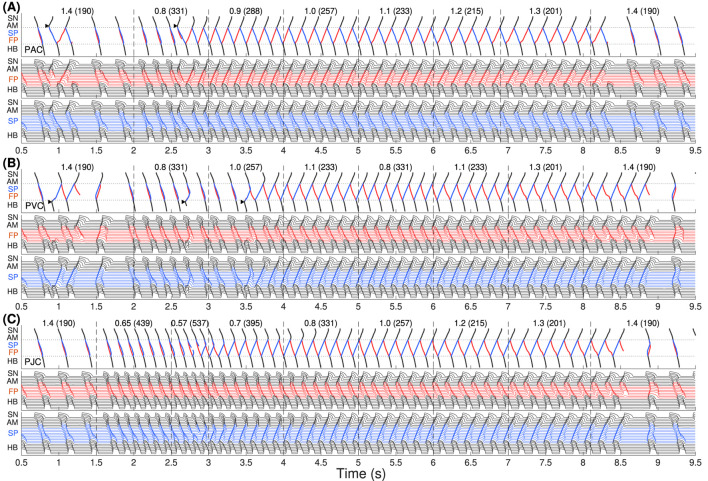
The same as in Figure 2 but for the second model variant with ${\text{rERP}}_{SP}<$ ${\text{rERP}}_{FP}$ . **(A)** Onset of slow-fast AVNRT with PAC at the enhanced sympathetic tone. At γ=1.4 , only slow-fast echo beats appeared. **(B)** Onset of the fast-slow form of AVNRT with PVC at the enhanced sympathetic tone. At γ=1.4 , only fast-slow echo beats appeared. **(C)** Similar to Figure 2, a brief burst of very strong sympathetic tone (equivalent to PJC) induced fast-slow form of AVNRT. In all cases **(A–C)**, spontaneous termination of the oscillations took place at increasing vagal tone (γ≥1.4) .

**FIGURE 7:**
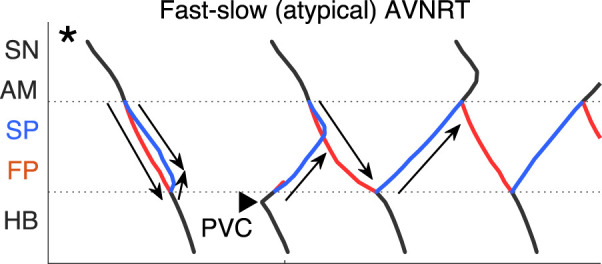
Scheme of the fast-slow AVNRT onset with PVC in the second model variant (from Figure 6B). Arrows show the direction of conduction via pathways. Arrowhead denotes the place and moment of premature stimulation. Asterisk indicates normal conduction.

**FIGURE 8:**
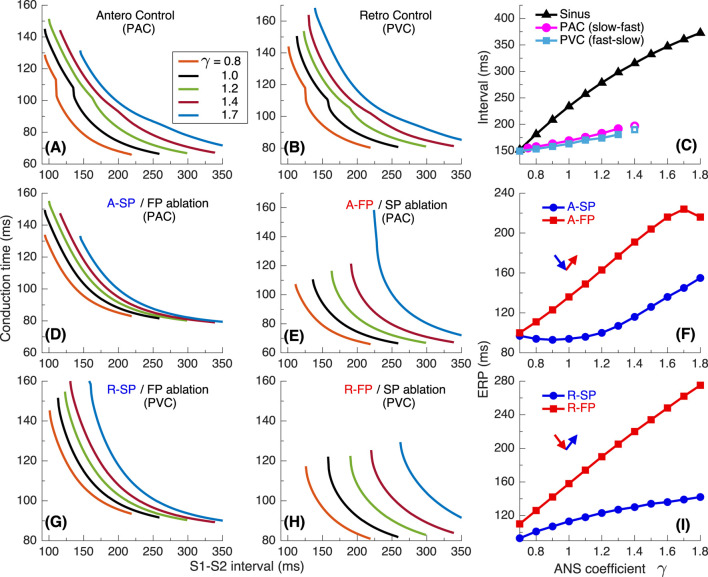
Conduction characteristics using S1S2 stimulation protocol for the second model variant. **(A)** Control case with atrial pacing (PAC). **(B)** Control case with His bundle pacing (PVC). **(C)** Dependence of sinus rhythm and AVNRT intervals of PAC and AV intranodal (PJC) origin on the coefficient γ. Empty markers correspond to echo beats. **(D, E)** - anterograde (A-) conduction times for SP (FP ablation) and FP (SP ablation), and their ERPs **(F)**; **(G, H)** - retrograde (R-) conduction times for SP and FP, and their ERPs **(I)**.

**FIGURE 9:**
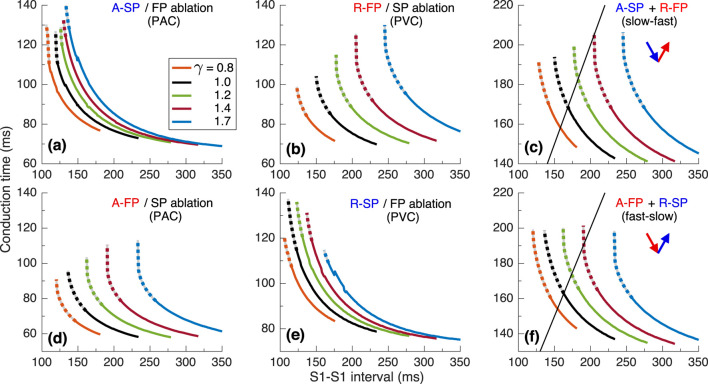
Conduction characteristics using S1S1 stimulation protocol for the second model variant. **(A, B)** - anterograde SP and retrograde FP conduction times and their sum (C) versus pacing interval. **(D, E)** - anterograde FP and retrograde SP conduction times and their sum (F) versus pacing interval. Unstable conduction is indicated by thick dotted parts on the conduction curves. Black straight lines in panels **(C, F)** Represent the AVNRT sustainability lines on which the stimulation interval equals the sum of the SP and FP conduction times.

Figures 6–9 present results with a similar simulation setup but for the second model variant with ${\text{rERP}}_{SP}\;<$ ${\text{rERP}}_{FP}$ in the entire range of γ .

In Figure 6A with atrial pacing we observed a similar situation as in Figure 2A, but spontaneous termination of slow-fast AVNRT occurred earlier at lower γ=1.4 . However, His bundle pacing gave different results (Figure 6B). First, a few fast-slow echo beats were initiated with γ=1.4 . Then, increasing γ from low values, we managed to induce fast-slow AVNRT only at γ≥1.0 . Sustained oscillations in the AV ring continued until γ reached 1.4. It was also possible to induce a fast-slow form of AVNRT with PJC by briefly decreasing γ to 0.57 in the same way as for the first model variant. In this case, the oscillations persisted with increasing γ up to 1.4 (Figure 6C).

Figure 7 demonstrates on the ladder diagram the details of the onset of fast-slow AVNRT with His bundle pacing (PVC). The beginning of the oscillations was facilitated by a subsequent sinus impulse, similar to the situation with PJC-originated fast-slow AVNRT shown in Figure 3B.

The anterograde S1S2 conduction curves in Figures 8A,D,E and the relationship of anterograde ERPs between SP and FP (Figure 8F) remained similar to those of the first model variant (Figures 4A,D,E,F). However, in contrast to Figure 4B, noticeable transitions of the conduction from FP to SP appeared on the retrograde control curves (Figure 8B). They became sharper with decreasing γ , due to the increase of ${\text{rERP}}_{FP}$ introduced in this model variant and reflected in Figure 8I with relatively wide induction window for fast-slow AVNRT.

Figure 9 shows the conduction characteristics using S1S1 stimulation protocol similar to that shown in Figure 5. As seen from Figures 6A, 9C, the sustainability of slow-fast AVNRT persisted only at γ<1.4 , that is, in the narrower range of γ than in the case of first model variant (Figure 5C). In the cases of His bundle pacing (PVC, Figure 6B) and PJC (Figure 6C) the upper sustainability limit for fast-slow AVNRT was also γ<1.4 (Figure 9F). It should be noted that with His bundle pacing at γ=0.8 , neither AVNRT nor echo beats were induced (Figure 6B), while fast-slow AVNRT initiated at higher γ values persisted when γ was temporarily reduced to 0.8.

### 3.3 The third model variant

**FIGURE 10:**
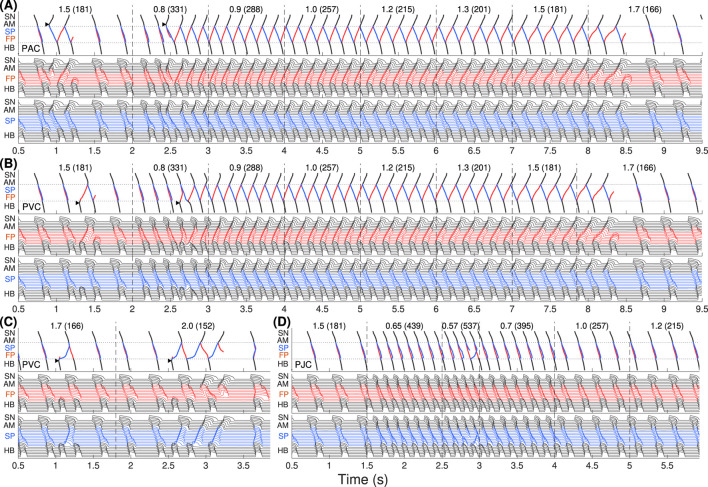
The same as in Figure 2 but for the third model variant. Onset of slow-fast AVNRT with PAC **(A)** and with PVC **(B)** at enhanced sympathetic tone, and spontaneous termination of the oscillations at increasing vagal tone at γ≥1.7 . With atrial and His bundle stimulation at γ=1.5 only slow-fast echo beats appeared. **(C)** Fast-slow echo beats occurred with PVC at highly predominant parasympathetic tone (γ≥1.7) . **(D)** In contrast to Figures 2, 6, a brief burst of very strong sympathetic tone did not induce any AVNRT.

**FIGURE 11:**
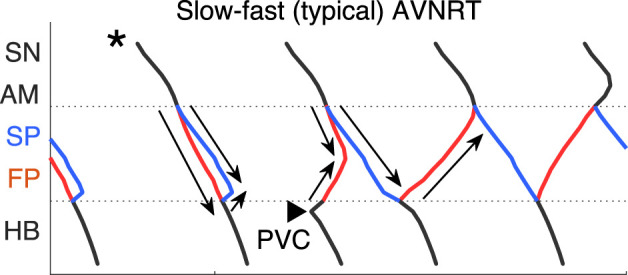
Scheme of slow-fast AVNRT onset with PVC in the third model variant (from Figure 10B). Arrows show the direction of conduction via pathways. Arrowhead denotes the place and moment of premature stimulation. Asterisk indicates normal conduction.

**FIGURE 12:**
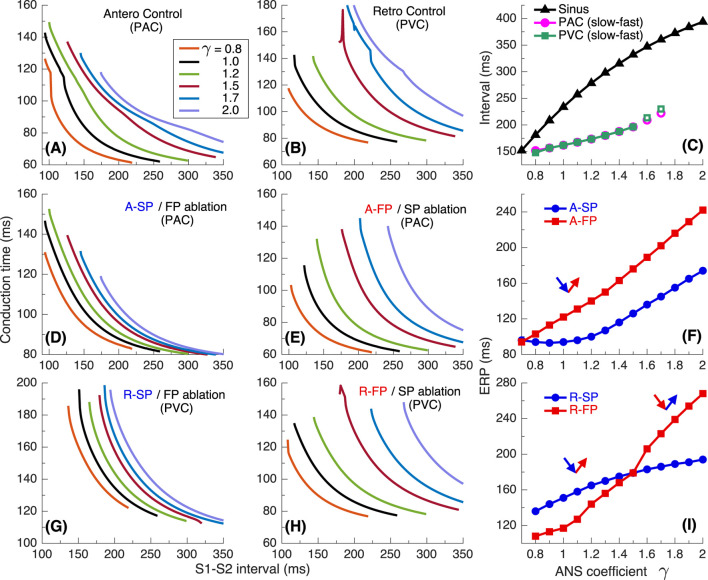
Conduction characteristics using S1S2 stimulation protocol for the third model variant. **(A)** Control case with atrial pacing (PAC). **(B)** Control case with His bundle pacing (PVC). **(C)** Dependence of sinus rhythm and AVNRT intervals of PAC and AV intranodal (PJC) origin on the coefficient γ. Empty markers correspond to echo beats. **(D, E)** - anterograde (A-) conduction times for SP (FP ablation) and FP (SP ablation), and their ERPs **(F)**; **(G, H)** - retrograde (R-) conduction times for SP and FP, and their ERPs **(I)**.

**FIGURE 13:**
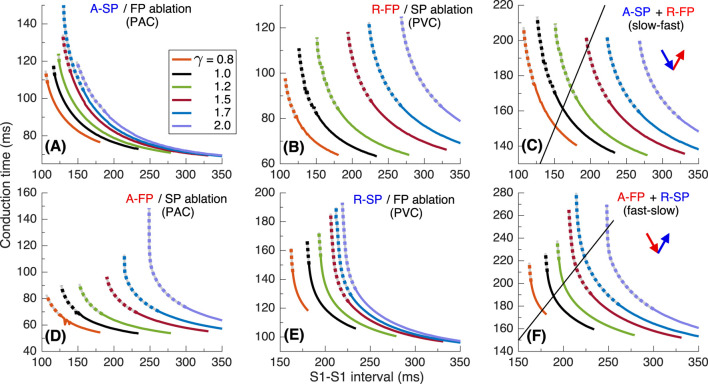
Conduction characteristics using S1S1 stimulation protocol for the third model variant. **(A, B)** - anterograde SP and retrograde FP conduction times and their sum (C) versus pacing interval. **(D, E)** - anterograde FP and retrograde SP conduction times and their sum (F) versus pacing interval. Unstable conduction is indicated by thick dotted parts on the conduction curves. Black straight lines in panels **(C, F)** represent the AVNRT sustainability lines on which the stimulation interval equals the sum of the SP and FP conduction times.

Figures 10–13 present simulation results obtained with the third model variant in which we set the relationship ${\text{rERP}}_{SP}>$ ${\text{rERP}}_{FP}$ at enhanced sympathetic tone and its inversion ( ${\text{rERP}}_{SP}<$ ${\text{rERP}}_{FP}$ ) at normal condition and enhanced vagal tone.

The situation with slow-fast AVNRT induction (Figure 10A) looks the same as in Figures 2A, 6A due to the similarity of anterograde conduction and refractory curves in panels (D)–(F) of Figures 4, 8, 12. However, with His bundle pacing (PVC, Figure 10B) we obtained the same typical slow-fast AVNRT form as with atrial pacing (PAC, Figure 10A). At γ=1.5 , both slow-fast echo beats and AVNRT were initiated depending on preceding conditions. We also managed to induce some fast-slow echo beats at enhanced parasympathetic tone with γ=1.7−2.0 (see Figure 10C).

In contrast to the first and second model variants, in the third variant, no AVNRT was induced with brief bursts of very strong sympathetic tone (Figure 10D). In Figure 11 the details of the onset of slow-fast AVNRT with His bundle pacing (PVC) are shown on ladder diagrams.

According to the setup of the third model variant, the retrograde FP and SP ERP curves shown in Figure 12I intersect at γ≃1.5 , which suggests induction of slow-fast AVNRT in the γ<1.5 range, and possible induction of fast-slow AVNRT at γ>1.5 . The peculiarity of the point γ=1.5 is reflected in the unusual form of retrograde control and FP conduction curves in their leftmost points in Figures 12B,H.

Results using S1S1 stimulation are demonstrated in Figure 13. Sustained slow-fast AVNRT can exist in the range γ≤1.5 (Figure 13C). The fast-slow form can exist within the entire range of γ which is supported by intersections of the AVNRT sustainability line with the whole set of conduction curves (Figure 13F), but it should be unstable with predominant parasympathetic tone at γ≥1.5 .

## 4 Discussion

Using our compact multifunctional model of rabbit AV node, we simulated the effect of ANS on the behavior of AVNRT. Incorporating a single ANS coefficient γ allowed the introduction of the combined effect of sympathetic and parasympathetic activity into the basic AV node model through the modulation of the refractoriness of model cells. A decrease in γ increases sympathetic tone, diminishes parasympathetic tone, and leads to a reduction in AV nodal conduction time and nodal refractory period in accordance with the results of electrophysiological studies (Morady et al., 1988; CossÃº et al., 1997). On the other hand, parasympathetic activity dominates with increasing γ and has opposite effects on AV nodal conduction time and refractory period (Martin, 1977). The approach is somewhat similar to one used in the work from Plappert et al. (Plappert et al., 2022). Still, our model utilizes a one-dimensional reduced-order reaction-diffusion system composed of 32 model cells and application of a single ANS control coefficient.

The validity of our approach to the incorporation of ANS control into our rabbit AV node model and obtained results are supported by the following clinical and experimental observations.

Clinical studies suggest that sustained typical slow-fast AVNRT episodes are preceded by an increase in sympathetic tone (Nigro et al., 2010). Accordingly, for the induction of AVNRT in most cases, we set γ to a small value (0.80–1.0, corresponding to enhanced sympathetic tone) and obtained slow-fast AVNRT (Figures 2A, 6A, 10A).

The atypical fast-slow AVNRT form appeared in our simulation in rare cases: in the case with PJC (Figures 2C, 6C), occurring primarily in children and postoperative patients (Chen et al., 2015), in the case with PVC (Figure 6B) with ${\text{rERP}}_{SP}<$ ${\text{rERP}}_{FP}$ (Sung et al., 1978), and in the even more marginal case with PVC (Figure 10C) at high vagal tone (Chiou et al., 2003). These results are supported by the clinical fact of a significant predominance of the typical slow-fast form of AVNRT over the atypical fast-slow one (Katritsis et al., 2015b).

According to Katritsis et al. (2015a), under certain conditions, the difference in retrograde ERPs between SP and FP can become inverted with variation of the ANS state (Figure 12I), resulting in both slow-fast and fast-slow AVNRT forms can occur in the same subject. In Figure 10A, typical slow-fast AVNRT appeared during predominance of sympathetic tone in γ≤1.5 range. On the other hand, the atypical fast-slow echo beats were induced during enhanced vagal tone (γ>1.7) (Figure 10C). The onset of AVNRT throughout periods of increased vagal tone, such as during sleep, has been observed in clinical practice (Chiou et al., 2003).

While the onset of AVNRT is mainly observed during enhanced sympathetic activity, a possibility exists for tachycardia or reciprocating beats induction with increased vagal tone due to the widening of the induction window (Chiou et al., 2003; Sinkovec et al., 2011). With increasing parameter γ and decreasing sinus rate, the inducibility of slow-fast AVNRT by atrial extrastimuli strengthened in all model variants (Figures 4F, 8F, 12F). We also observed the same effect of ANS modulation on the fast-slow AVNRT inducibility with His bundle stimulation for the second model variant (Figure 8I).

The *onset* of AVNRT requires four factors: (A) the presence of at least two functional pathways in the AV node, (B) a specific ANS state that ensures the appropriate refractoriness of the pathways and conduction delays in them, (C) a difference in ERPs between the slow and fast pathways, and (D) the presence of a premature atrial or His bundle (ventricular) stimulus delivered at the proper time. The latter condition is not required when AVNRT originated within the AV node (PJC). Induction of AVNRT in response to a brief burst of enhanced sympathetic tone accompanied by fast sinus rhythm or atrial pacing was reported in patients (Chiou et al., 2003). This phenomenon was also observed in our simulations (Figures 2C, 6C).

The *sustainability* of AVNRT is determined by the coincidence of the total duration of anterograde and retrograde conduction in slow and fast pathways with the AVNRT cycle length dependent on the state of the ANS (panels (C) and (F) in Figures 5, 9, 13). If the AVNRT sustainability condition is not satisfied but the conditions for its onset mentioned in the previous paragraph are met, a few echo beats may occur (Figures 6B, 8I).

As seen in Figures 2A, 6B, 10A,B, at the same coefficient γ all kinds of behavior may be observed - a few echo beats, sustained tachycardia, or its termination, depending on the preceding activity. Thus, from the nonlinear dynamics point of view, the sustainability criteria appear to be a basin of attraction (Izhikevich, 2006), when external stimuli at a particular set of initial conditions either lead to persistent oscillations within the AV node ring, which may be accompanied by cycle length variability (Tamura et al., 2020), or to fading echo beats. Studying the nonlinear nature of the AVNRT sustainability is no doubt a very interesting and exciting topic requiring separate dedicated investigation.

The shapes of anterograde control conduction curves, obtained using the S1S2 protocol with varying coefficient γ exhibit more or less pronounced tilting and bending at the point of conduction switching between the fast and slow pathways (Figures 4A, 8A, 12A) (Reid et al., 2003; Ryzhii and Ryzhii, 2023a). The bending and the nodal conduction discontinuity became more pronounced with smaller γ . The discontinuity position hardly observed at large γ , shifted toward a shorter S2 coupling interval due to a significant decrease of ${\text{aERP}}_{FP}$ . The smooth and discontinuous anterograde conduction curves were experimentally demonstrated in rabbits (Reid et al., 2003; Zhang, 2016) and in humans (Sung et al., 1978). The nodal conduction discontinuities that appeared in some retrograde control conduction curves with PVCs in the second model variant (Figure 8B) and in the third model variant (Figure 12B) were also observed in patients (Wu et al., 1977; Sung et al., 1978) due to the prevalence of ${\text{rERP}}_{FP}$ over ${\text{rERP}}_{SP}$ (Figures 8I, 12I).

In most cases of anterograde and retrograde conduction, vagal modulation affected the ERPs of the FP more strongly than the ERPs of the SP (see panels (F) and (I) in Figures 4, 8, 12), as quantitatively demonstrated by Chiou et al. (2003).

Applying S1S1 stimulation protocols, we observed some cases of nodal conduction alternans with variation of conduction time from beat to beat (Sun et al., 1995; Garfinkel, 2007), which are reflected in some bumps on conduction curves in Figures 5A,E, 9A,E, 13B,D.

The *spontaneous termination* of AVNRT occurs due to increased refractoriness and nodal conduction delays (Plappert et al., 2022), resulting from enhanced vagal tone, which is utilized in Valsalva maneuvers and pharmaceutical treatments (Appelboam et al., 2015; Xiao et al., 2024). When the combined delays of anterograde and retrograde conduction in the SP and FP become unequal to the pacing interval, the condition for persistent AVNRT is no longer met (panels (C) and (F) in Figures 5, 9, 13). In the vast majority of cases, spontaneous termination of AVNRT occurred when the conduction was blocked through FP regardless of the AVNRT form (Figures 2A,C, 6, 10A–C). This aligns with clinical observations regarding spontaneous AVNRT termination (Chiale et al., 2015).

The summary of different types of AVNRT and echo beats obtained in our simulations is given in Table 1, where S-F and F-S mean slow-fast and fast slow types, and asterisk denotes echo beats. In the table, “Pulse” type corresponds to the onset of AVNRT induced by either PAC or PVC, and “ANS tone change” type is related to the sustainability of the oscillations. As seen from Table 1, at enhanced parasympathetic tone (γ≥1.7) the reentrant activity decreases significantly and is represented only by echo beats. The data in the table includes cases of the echo beats occurrence with PAC not demonstrated in the ladder diagrams.

**TABLE 1:** Induction of different types of AVNRT in the three model variants.

	Induction type		γ=0.8	1.0	1.2	1.4/1.5	1.7	2.0
Var. 1	PAC	Pulse	S-F	S-F	S-F	S-F*	S-F*	S-F*
		ANS tone change	S-F	S-F	S-F	S-F	S-F*	-
	PVC	Pulse	-	-	-	F-S*	F-S*	F-S*
		ANS tone change	-	-	-	-	-	-
	PJC	ANS tone change	F-S	F-S	F-S	F-S	-	-
Var. 2	PAC	Pulse	S-F	S-F	S-F	S-F*	-	-
		ANS tone change	S-F	S-F	S-F	S-F*	-	-
	PVC	Pulse	-	F-S	F-S	F-S*	F-S*	F-S*
		ANS tone change	F-S	F-S	F-S	F-S*	-	-
	PJC	ANS tone change	F-S	F-S	F-S	F-S*	-	-
Var. 3	PAC	Pulse	S-F	S-F	S-F	S-F*	-	-
		ANS tone change	S-F	S-F	S-F	S-F	S-F*	-
	PVC	Pulse	S-F	S-F	S-F	S-F*	F-S*	F-S*
		ANS tone change	S-F	S-F	S-F	S-F	S-F*	-
	PJC	ANS tone change	-	-	-	-	-	-

Asterisk denotes echo beat(s).

While there are differences in the detailed electrophysiology of the atrioventricular (AV) nodes between rabbits and humans, particularly regarding the origin of AV nodal pacemaking (Mazgalev et al., 2001), the general behavior is similar across mammals. Therefore, experiments and simulations conducted on animals remain important (Bartolucci et al., 2024). We believe that the assumptions underlying the proposed model, the simulation results obtained, and the conclusions drawn correspond qualitatively, if not in perfect detail, to the actual processes occurring in the human heart.

## 5 Limitations

While a simplified approach views the sympathetic and parasympathetic branches of the autonomic nervous system (ANS) as having opposite effects, a contemporary perspective recognizes that the sympathetic branch is responsible for quick mobilization responses, whereas the parasympathetic branch serves as a gradually activated damping system. Our phenomenological model adopts this simplified view to provide a general macroscopic description of the ANS control over the cardiac conduction system. Though a uniform coefficient makes the ANS control implementation simple, it complicates studying the effects of medications on the sympathetic and parasympathetic limbs of the ANS separately.

The second limitation of the current version of the rabbit conduction system model is the absence of heart rate variability (HRV). HRV is also governed by the balance between parasympathetic and sympathetic tones of the ANS (Guzzetti et al., 2005; Rovere et al., 2020), and impacts the cardiac conduction system, particularly the sinus pacemaker, over the relatively long term (24 h). However, our study specifically focuses on the effects of the ANS over a shorter time scale. The slow variation in the sinus rate has minimal impact on AVNRT since the rate in the latter is consistently higher, and sinus rhythm is effectively overdriven [panels (C) in Figures 4, 8, 12]. Consequently, we can disregard the influence of HRV in our analysis.

The third limitation is that we applied the ANS control coefficient γ to SP and FP on the same scale, which led to some differences in their response. In contrast, in reality, the degree of influence of ANS on the AV nodal pathways may differ.

Finally, the current structure of the AV node model includes only one slow pathway. The atypical slow-slow form of AVNRT was left out of our model. This form of AVNRT is observed in a small percentage of cases in both humans (Katritsis et al., 2015b) and rabbits (Patterson and Scherlag, 2003). However, due to the limited data available on rabbits, incorporating a second slow pathway into the AV node model presents challenges. This remains a topic for future development of the AV node model.

## 6 Conclusion

In this work, we extended the functionality of our previously developed model of the rabbit cardiac conduction system based on the Aliev-Panfilov cardiac cell model by incorporating control from the autonomic nervous system. The control is accomplished by altering cell refractoriness using a single coefficient, which changes the conduction delays in the AV nodal pathways and intrinsic frequency of pacemaker cells. The influence of the autonomic nervous system extends to the model cells from the sinus node to the penetrating bundle.

Using the modified model, we studied conditions for the onset, sustainability, and spontaneous termination of typical slow-fast and atypical fast-slow AVNRT forms. The conditions for the onset and sustainability of AVNRT can occur independently in various combinations. The difference in effective refractory periods between slow and fast pathways and the state of the autonomic nervous system determine the type of AVNRT and its sustainability with both atrial pacing and His bundle pacing.The updated computationally lightweight but detailed model of rabbit cardiac conduction system with dual AV nodal pathways is suitable for studying physiological mechanisms of various forms of AVNRT. Inclusion of autonomic nervous system control into the model provides more lifelike functionality and allows realization of various situations that are nearly impossible to reproduce in animal or human experiments. Our model could also serve as an educational tool to help students and practitioners visualize and understand the dynamic and complex interactions leading to AVNRT.

## Data Availability

The MATLAB code used in this study can be found in the GitHub repository https://github.com/mryzhii/rabbit-AVNRT and Zenodo repository https://zenodo.org/records/14604033.
